# Dysregulated maternal and newborn fatty acid, sugar and amino acid metabolism associated with high birth weight

**DOI:** 10.1038/s41366-025-01775-9

**Published:** 2025-04-17

**Authors:** Chenyu Qiu, Jiawen Liao, Roya Gheissari, Claire Li, Anika Kapai, David V. Conti, Dean P. Jones, Theresa M. Bastain, Carrie V. Breton, Zhanghua Chen

**Affiliations:** 1 Department of Population and Public Health Sciences, Keck School of Medicine of the University of Southern California, Los Angeles, CA USA; 2 Department of Medicine, Keck School of Medicine of the University of Southern California, Los Angeles, CA USA; 3 Department of Medicine, Emory University, Atlanta, GA USA

**Keywords:** Preventive medicine, Paediatrics

## Abstract

**Objective:**

This study aims to find maternal and neonatal metabolomic signatures that contribute to the adverse birthweight outcomes including abnormally high and low birth weight. We also investigated the role of metabolomic signatures in the associations of maternal risk factors such as parity and gestational weight gain with adverse birthweight outcomes.

**Methods:**

Ninety-six pregnant women and their newborns from the MADRES prospective cohort were studied. Maternal serum at third trimester and newborn cord blood were assayed for untargeted metabolomics using mass-spectrometry. Metabolome-wide association analysis was conducted to assess maternal and newborn metabolomic features association with birth weight Z-score, followed by network analysis of maternal and newborn metabolomics. Lastly, the contribution of maternal and newborn metabolomics to associations between maternal risk factors and newborn birthweight was assessed.

**Results:**

Maternal gestational weight gain and parity were positively associated with newborn birthweight. Maternal glucose and branched-chain amino acid metabolism pathways and newborn’s fatty acid, glucose metabolism and C21-steroid hormone biosynthesis were significantly enriched with high birth weight Z-score. Dysregulation in these pathways linked maternal factors such as gestational weight gain and parity with high birth weight Z-score.

**Conclusion:**

Our findings indicate that altered maternal sugar and energy metabolism, newborn sugar and amino acid metabolism, and newborn C21-steroid hormone biosynthesis were associated with high birth weight. Dysregulated metabolism in pregnant women and newborn may contribute to the pathophysiological mechanisms linking maternal excessive gestational weight gain and multiparity with high birth weight.

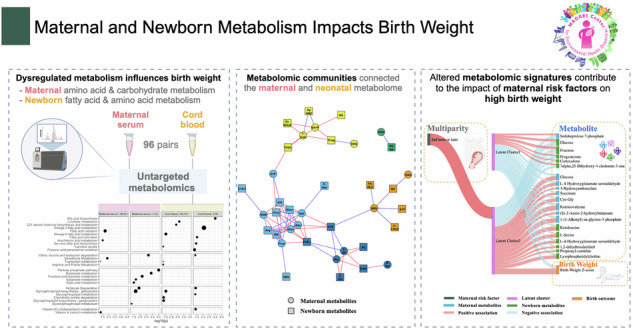

## Introduction

Both small for gestational age (SGA) and large for gestational age (LGA) are risk factors for obesity, hypertension, and metabolic syndrome for children in later life [1–5]. Maternal metabolism during pregnancy is known to affect placental nutrient transportation and fetal metabolism. Using metabolomics methods, previous studies identified several metabolites and altered metabolic pathways that inform pathophysiological mechanisms causing variation in birth weight. For example, several studies investigating cord blood metabolomics have reported positive associations between birth weight and cord blood concentrations of vitamin A and lysophosphatidylcholines, and both low and high birth weight have been associated with altered levels of amino acids, acylcarnitines, tryptophan metabolites, and fatty acids [6–9]. Other studies analyzing maternal metabolites during the third trimester have found positive associations between newborn birth weight and maternal levels of fructose, citrate, and several lipid-related metabolites such as acylcarnitines and triglycerides [10, 11]. Additionally, studies of pregnant women showed that concentrations of branched-chain amino acids and fatty acids in maternal blood samples collected during second and third trimesters were associated with fetal growth, newborn size, and infant obesity [5, 10, 12, 13].

However, most previous studies have been focusing on either maternal or newborn metabolomics, with limited knowledge on how maternal metabolism and newborn metabolism jointly impact birth weight. To our knowledge, only one human study of 16 mother-newborn pairs has investigated both maternal and newborn metabolomics. They found that increased levels of glutamine in both maternal blood at delivery and cord blood were associated with preterm and very low birth weight, albeit this study analyzed maternal and newborn metabolomics separately. Meanwhile, low-density lipoproteins and pyruvate concentrations were observed to be higher in maternal blood and lower in cord blood, in association with very low birth weight [14]. Since maternal metabolism influences fetal and newborn metabolism throughout the pregnancy, it is valuable to investigate the interactions between maternal and newborn metabolism, as well as the mechanisms by which altered metabolism in mother and newborn affects birth weight, especially among an underrepresented population with higher risk of adverse birth outcomes such as LGA and childhood obesity [15, 16]. In this study, we analyzed both maternal and newborn untargeted metabolomics in 96 mother-newborn pairs from a largely Hispanic cohort with low socio-economic status and higher risk for small and large gestational age [17]. Subsequently, we also examined how these adverse birth outcomes were associated with the mother-newborn metabolomic network as well as with various maternal characteristics.

## Materials/subjects and methods

### Study recruitment and study design

Participants included in this analysis came from the Maternal And Developmental Risks from Environmental and Social Stressors (MADRES) pregnancy cohort. Details of the MADRES study design and recruitment have been published previously [17]. Briefly, the MADRES cohort is an ongoing prospective pregnancy cohort that started in 2015 and mainly consists of lower-income Hispanic pregnant women of age ≥18 from Los Angeles, California. The exclusion criteria included HIV positive status; physical, mental, or cognitive disability that prevents participation or providing informed consent; current incarceration; or multiple gestation. Health questionnaires were administered at early and late pregnancy. Among participants who completed health questionnaires during pregnancy, 96 mother–newborn pairs who contributed their newborn cord blood samples and 3rd trimester maternal blood samples were included in the final analysis of this study. Of the 96 women, 74 were enrolled before 20 weeks of gestation (regular entry) and 22 women were enrolled between 20 weeks and 30 weeks of gestation (late entry). Written informed consent and HIPAA authorization to access medical records of pregnant women and their newborns were obtained at study entry and signed by the women before their enrollment. The study was approved by the University of Southern California’s Institutional Review Board.

### Main outcome of birth weight

Birth weight was obtained from medical records, and the 2017 US national reference for singleton birth weight [18] was used for birth weight percentiles. Birth weight in grams was further transformed into sex-specific weight-to-gestational age Z-score (birth weight Z-score). Newborns with birth weight Z-score <10th percentile were categorized as SGA, newborns with birth weight Z-score >90th percentile were categorized as LGA, and the remaining newborns were categorized as appropriate for gestational age.

### Untargeted metabolomics analysis

High-resolution untargeted metabolomic profiles were analyzed through well-validated methods [19, 20]. The 96 pairs of cord blood plasma and 3rd-trimester maternal serum samples were analyzed in triplicates by hydrophilic interaction liquid chromatography (HILIC) with positive electrospray ionization (ESI) and C18 hydrophobic reversed-phase chromatography with negative ESI. Raw data was extracted by apLCMS [21] and was modified using xMSanalyzer [22]. Unique detected ions, which were characterized by m/z, retention time, and ion abundance, were referred to as metabolomic features. Batch effects were corrected using ComBat [23]. Metabolomic features that were less than 50% detected across samples were excluded from the final analysis. More details about data imputation and transformation are described in Supplemental Material and Fig. S1. After the data quality control, the final statistical analysis included 8242 features from the HILIC positive mode analysis and 5071 features from the C18 negative mode analysis using cord blood samples, as well as 8186 features from the HILIC positive mode analysis and 5133 features from the C18 negative mode analysis using maternal serum samples.

### Covariates

Maternal characteristics including pre-pregnancy weight, Hispanic race/ethnicity, smoking history, marital status, education level, and parity were collected by self-reported questionnaires from women at their first study visit. Maternal height was measured by a stadiometer at the first and third trimester study visits. Pre-pregnancy body mass index (BMI, kg/m^2^) was calculated as pre-pregnancy weight (kg) divided by the square of height (m^2^). Lifetime cigarette smoking was determined by questionnaire responses in each trimester. Maternal age at delivery was calculated based on delivery date and women’s birth date. Maternal weight measurements during pregnancy were obtained from prenatal healthcare medical records and the study measured weight at in-person visits. Gestational weight gain was calculated by subtracting the self-reported pre-pregnancy weight from the last weight recorded at the third-trimester visit. Other newborn demographics and delivery information including sex and Hispanic race/ethnicity, and delivery method were retrieved from questionnaire and electronic medical records [17]. We used a hierarchy of methods to calculate and standardize gestational age at birth for newborns [24]. Gestational age at the time of sample collection in weeks was derived by deducting the difference in the number of weeks between the infant’s date of birth and the date of the sample collection from the gestational age in weeks at the time of birth. Sample processing time was calculated as the start time of sample processing deducted by the time at sample collection. Daily total calorie intake, which was computed automatically from the web-based National Cancer Institute’s Automated Self-Administered 24-hour Dietary Assessment Tool (ASA24) [25], was derived by averaging from multiple ASA24 dietary recalls during pregnancy.

### Statistical analysis

Associations of maternal health factors including pre-pregnancy BMI, gestational weight gain, gestational diabetes mellitus, hypertension, parity, daily total calorie intake, and smoking history, with birth weight Z-score as a continuous variable were investigated adjusting for covariates such as enrollment groups (regular vs. late entry), recruitment sites, maternal age at delivery, newborn race/ethnicity, household income, marital status, and education level using linear regression models. Then, metabolome-wide association analysis (MWAS) was conducted using linear regression models to investigate associations of either maternal or newborn metabolomic feature with birth weight Z-score adjusting for enrollment groups (early vs. late entry), recruitment sites, maternal age at delivery, household income ($30,000 or below, more than $30,000, or unknown), marital status (single or divorced, living together, married, or unknown), education level (less than 12th grade, completed high school, college or technical school or above, or unknown), newborn Hispanic race/ethnicity (yes, no, or unknown), lifetime smoking history (ever or never) and sample processing time (within 3 hours or longer than 3 hours). The Benjamini-Hochberg procedure [26] was used to evaluate the false discovery rate (FDR) for multiple testing.

Mummichog pathway enrichment analysis with 10,000 permutations was used to identify significant metabolic pathways and provide predicted feature annotation [27]. For statistically significant pathways (*p* < 0.05), chemical identities of metabolomic features involved in these pathways were further confirmed by comparison to a lab-developed reference of 467 chemical compounds using MS^2^ spectra [28]. The error tolerance was 5ppm for m/z and 30 s for retention time. Only features matched to the compound reference were presented as metabolites with confirmed identity according to the Metabolomics Standards Initiative (MSI) level 1 criteria [29]. Metabolomic features that were not included in the reference were annotated by Mummichog.

xMWAS integrated network analysis [30] was then performed to investigate the intertwined mother-newborn metabolomics network. Metabolites from both mothers and newborns that were involved in Mummichog pathways and associated with birth weight Z-score were included in the xMWAS analysis adjusting for the same covariates as those previously controlled for in the linear regression models. Correlation threshold ≥0.4 and significance level of *p* < 0.05 were used to select potentially important connections between maternal and newborn metabolites. We used the degree weight centrality measure (DWCM: range from 0 to 1) to assess the importance of nodes in the entire network. Based on the sum of a node’s absolute weights of connection, high DWCM indicated stronger connections in the network.

We further investigated whether maternal and newborn metabolomics contribute to the associations found between maternal health factors and newborn birth weight using the latent unknown clustering with integrated data (LUCID) [31] (using R package *“LUCIDus”*) analysis adjusting for covariates. The LUCID analysis used data for maternal health factors and metabolite signatures as well as newborn metabolites to identify clusters of mother-newborn pairs that differed by newborn birth weight. The optimal number of latent clusters (*K*) was identified by the model with the lowest Bayesian information criterion (BIC) and two clusters were identified in this study. The cluster assignment of each mother–newborn pair was determined by the posterior probabilities for the two latent clusters, and we used 0.5 as the cutoff for posterior probabilities. Two-sample t-test was used to identify key maternal and newborn metabolites that have different intensities between the two clusters. Fisher’s exact test was used to verify the associations between latent clusters and birth weight categories in this supervised clustering analysis.

## Results

Maternal and newborn characteristics are presented in Table 1. The 96 MADRES mothers ranged from age 18.8 to 42.7 years old at delivery, 49.0% of the 96 mothers had a household income less than $30,000, 78 (81.3%) women were Hispanic, 30 (31.3%) women had an education level lower than high school diploma, and 61 (63.5%) women had two or more children. There were 66 (68.8%) women with overweight or obesity before pregnancy. 52 (54.2%) newborns were female and 44 (45.8%) were male. The mean ± SD birth weight was 3.36 ± 0.49 kg, and 13 (13.5%) newborns were classified as LGA and 8 (8.3%) newborns were SGA. The mean ± SD gestational age was 39.13 ± 1.44 weeks, 8 (8.3%) were preterm births with gestational age less than 37 weeks, 87 (90.6%) were term births between 37 and 42 weeks of gestation, and only 1 (1.1%) was post-term newborn (after 42 weeks of gestation). Table 1 also presented the associations and p-values between participants’ characteristics and newborn birth weight Z-score. Second-born or later-born newborns had higher birth weight compared to first-borns (*p* = 0.01) and greater maternal weight gain was associated with higher birth weight in newborn (*p* = 0.01). No other statistically significant associations were observed between maternal characteristics and newborn birth weight.

**Table 1:** Characteristics of 96 MADRES mother-newborn pairs and their associations with newborn birth weight Z-score^a^.

	Entire sample (*N* = 96)	Associations with Birth Weight Z-score	
	*N* (%)	*β* (95% CI)	*p* value
(A) Maternal characteristics			
Maternal age at delivery (year)	28.71 (27.58–29.84)^b^	−0.03 (−0.08–0.02)	0.21
Gestational weight gain^c^ (kg)	11.90 (10.44–13.36)^b^	0.05 (0.01–0.09)	**0.01**
Total calorie intake, kcal/d	1883 (1766–1999)^b^	−0.0002 (−0.0007–0.0003)	0.44
Hispanic Ethnicity			
No	17 (18)	—	—
Yes	78 (81)	—	—
Unknown	1 (1)	—	—
Household income			
$30,000 or below	47 (49)	REF	REF
More than $30,000	20 (21)	0.20 (−0.45–0.85)	0.55
Unknown	29 (30)	0.07 (−0.52–0.66)	0.81
Maternal education			
Less than 12th grade	30 (31)	REF	REF
Completed high school	33 (34)	−0.11 (−0.79–0.57)	0.74
College or technical school or above	31 (32)	0.02 (−0.70–0.74)	0.96
Unknown	2 (2)	−0.65 (−3.18–1.89)	0.61
Marital status			
Married	27 (28)	REF	REF
Living together	40 (42)	−0.32 (−0.95–0.30)	0.31
Single or divorced	23 (24)	−0.57 (−1.28–0.15)	0.12
Unknown	6 (6)	−1.02 (−2.89–0.85)	0.28
Lifetime cigarette smoking^d^			
Never used	64 (67)	REF	REF
Ever used	32 (33)	−0.08 (−0.65–0.49)	0.79
Pre-pregnancy BMI (CDC category)			
Underweight or normal weight	30 (31)	REF	REF
Overweight	31 (32)	−0.24 (−0.89–0.40)	0.46
Class 1 obese	24 (25)	0.09 (−0.67–0.86)	0.81
Class 2 or class 3 obese	11 (11)	0.3 (−0.66–1.26)	0.53
Parity			
First-born	31 (32)	REF	REF
Second-born or later	61 (64)	0.80 (0.19–1.41)	**0.01**
Unknown	4 (4)	1.51 (−1.13–4.15)	0.26
Glucose metabolism			
Normal	60 (63)	REF	REF
Glucose intolerant	23 (24)	−0.06 (−0.68–0.56)	0.84
GDM or chronic diabetes	13 (14)	0.14 (−0.60–0.88)	0.71
Hypertension			
Normal blood pressure	79 (82)	REF	REF
Hypertensive	17 (18)	0.06 (−0.63–0.74)	0.87
Delivery type			
Normal spontaneous vaginal delivery	64 (67)	—	—
Non-NSVDe	32 (33)	—	—
(B) Newborn characteristics			
Birth weight (kg)	3.36 (3.26–3.46)^b^	—	—
Gestational age at birth (week)	39.13 (38.84–39.42)^b^	—	—
Hispanic Ethnicity			
No	14 (15)	REF	REF
Yes	80 (83)	0.33 (−0.41–1.07)	0.38
Unknown	2 (2)	−0.65 (−3.18–1.89)	0.61
Sex			
Female	52 (54)	—	—
Male	44 (46)	—	—
Birth weight category^f^			
AGA (Appropriate for Gestational Age)	75 (78)	—	—
SGA (Small for Gestational Age)	8 (8)	—	—
LGA (Large for Gestational Age)	13 (14)	—	—

^a^Multivariable linear regression model was used to investigate the association of the main maternal and newborn characteristics with birth weight Z-score.

^b^These variables are presented as mean (95% confidence interval) rather than *N* (%).

^c^Gestational weight gain = the total weight gain during pregnancy period.

^d^Lifetime cigarette smoking = ever used cigarette before pregnancy and during pregnancy.

^e^NSVD = normal spontaneous vaginal delivery; Non-NSVD = planed C-section, unplanned/emergency C-section, vaginal birth after cesarean (VBAC), and vacuum-assisted vaginal delivery.

^f^Small for Gestational age (GA) is defined as <10th percentile for birth weight; Large for GA is defined as >90th percentile for birth weight.

Significant associations with *p * <  0.05 were bolded.

### Maternal and newborn metabolomics in association with birth weight Z-score

MWAS analyses suggested that 808 maternal serum metabolomic features and 737 newborn metabolomic features had statistically significant associations with birth weight Z-score (*p* < 0.05) (Fig. S3). Among maternal serum metabolomic features, 397 (49.1%) and 411 (50.9%) were positively and negatively associated with birth weight Z-score, respectively. Among newborn cord blood metabolomics, 411 (55.8%) and 326 (44.2%) were positively and negatively associated with birth weight Z-score, respectively.

**Fig. 1:**
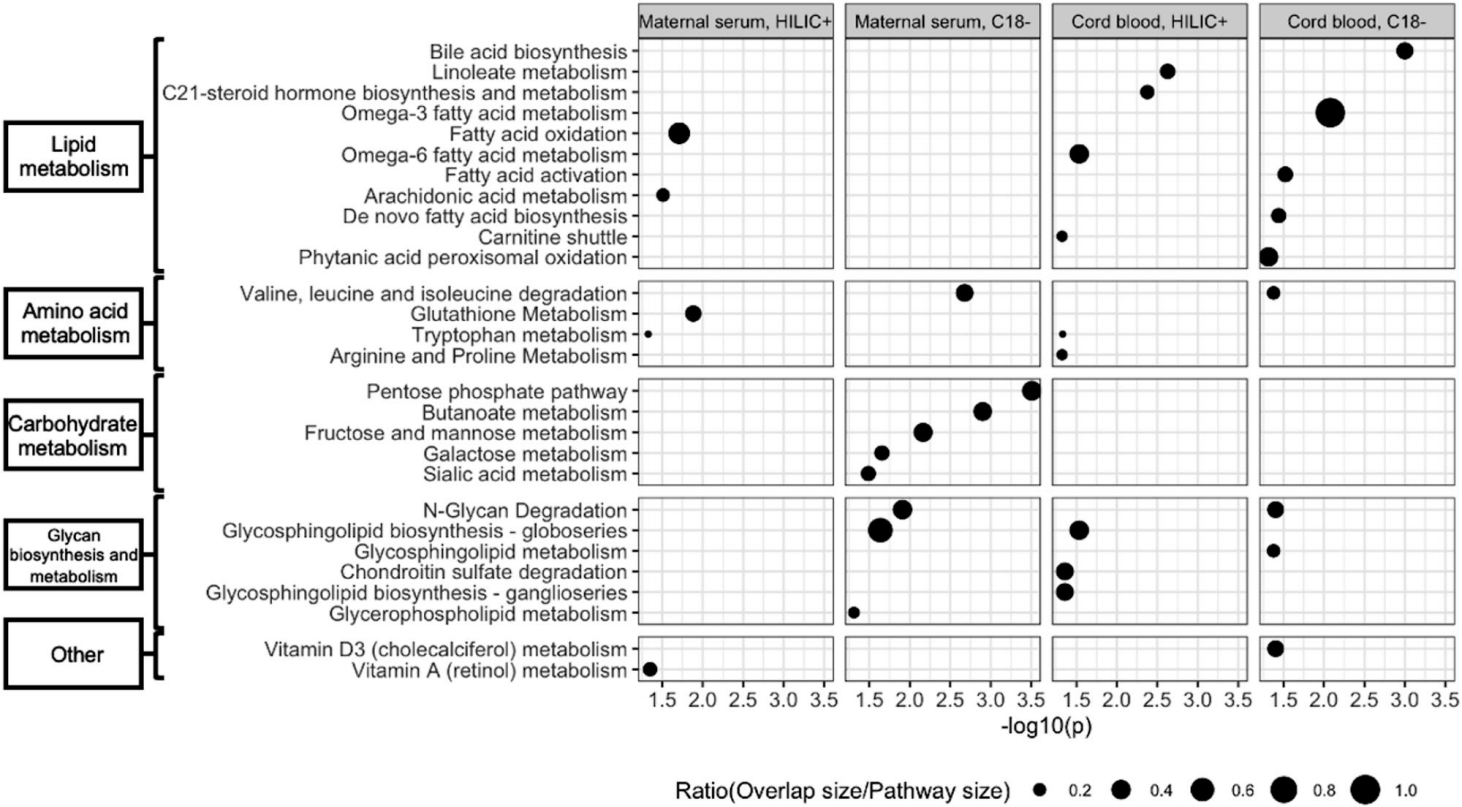
Metabolic pathways that were associated with birth weight revealed by the analysis of metabolomic features from 3rd-trimester maternal serum samples and cord blood plasma samples using both HILIC positive and C18 negative modes. The significance levels of Mummichog pathway enrichment analysis were presented on the horizontal scale.

Mummichog pathway enrichment analysis found that the most statistically significant pathways associated with birth weight included dysregulated branched-chain amino acid (BCAA) metabolism, pentose phosphate pathway, butanoate metabolism, and fructose and mannose metabolism in mothers in 3rd trimester and dysregulated bile acid biosynthesis, linoleate and omega-3 fatty acid metabolism, and C21-steroid hormone biosynthesis and metabolism in newborns (Fig. 1). Among all identified metabolic pathways that were associated with birth weight, Mummichog analysis provided tentative annotations for 32 maternal metabolomic features and 44 cord blood metabolomic features (Tables S1 and S2). We confirmed three maternal metabolomic features including fructose, glucose, and 2-deoxyglucose (the top three features in Table S1) and eight cord blood metabolomic features including progesterone, cortexolone, arachidic acid, oxovalerate/ketoisovalerate, ketoleucine/ketoisoleucine, fructose, 2-deoxyglucose, and glucose (the top eight features in Table S2) for their chemical identity by comparing the MS spectra to chemical standards.

### Integration of maternal and newborn cord blood metabolomics using xMWAS

To investigate the connection between maternal and newborn metabolomics, xMWAS network analysis was used to dissect 32 maternal and 44 newborn cord blood metabolomic features into 5 subnetworks that were tentatively annotated by Mummichog analysis (Fig. S4). Nine maternal metabolites and four newborn metabolites showed stronger connections in the entire network (DWCM > 0.2) (Table S3). The first subnetwork negatively connected a maternal isoleucine biosynthesis-related metabolite, (S)-2-aceto-2-hydroxybutanoate, with newborn fatty acids including arachidic acid and octadecenoate. The second subnetwork negatively correlated maternal glucose, fructose, and ketoisovalerate with newborn glucose, fructose, and L-arginine, and it positively correlated maternal glucose, fructose, and ketoisovalerate with newborn carnitines and newborn metabolites involved in branched-chain amino acid metabolism (oxovalerate/ketoisovalerate and ketoleucine/ketoisoleucine). The third subnetwork positively connected a maternal pentose phosphate pathway-related metabolite (sedoheptulose 7-phosphate) and a fatty acid oxidation intermediate (tetradecanoyl-CoA) with newborn bile acid metabolism, and newborn C21-steroid hormone biosynthesis and metabolism. The fourth subnetwork positively connected maternal metabolites involved in carbohydrate metabolism (2-deoxyglucose and L-ribulose) with newborn cortexolone, 2-deoxyglucose, and 1-pyrroline-2-carboxylate. Additionally, this subnetwork negatively associated maternal metabolites involved in carbohydrate metabolism with two newborn long-chain acylcarnitines.

### Maternal and newborn metabolomics linking maternal parity and gestational weight gain to higher birth weight

As previously presented, among the maternal characteristics, parity and gestational weight gain were the significant risk factors for high birth weight (Table 1). Therefore, LUCID analysis was used to integrate these maternal risk factors with key maternal and newborn metabolomic signatures, as well as to assess the joint association with birth weight.

**Fig. 2:**
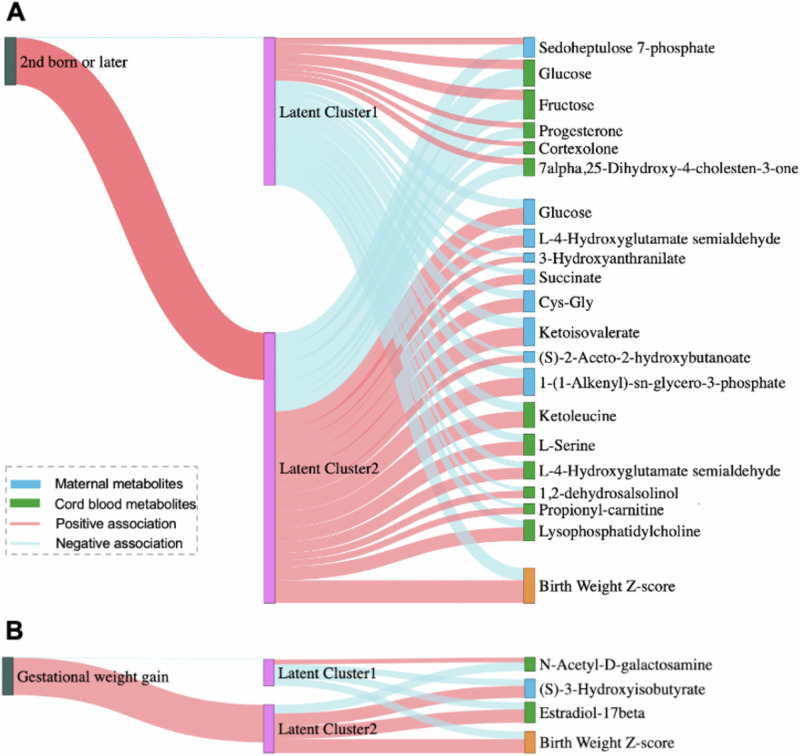
Integrative analysis for the effects of two maternal factors and metabolites on birthweight Z-score, using latent unknown clustering with integrated data. **A** shows the plot involved multiparous as the determinant factor (with first born as the reference) 9, maternal and 11 cord blood metabolites as mediators, and birth weight Z-score as the outcome. **B** presents the plot involved gestational weight gain as the determinant factor, 1 maternal and 2 cord blood metabolomic signatures as mediators, and birth weight Z-score as the outcome.

Among the 76 annotated metabolites associated with birth weight, 9 maternal metabolites and 11 cord blood metabolites had significant associations with parity (all *p*-values < 0.05) (Table S4). Then, the 9 maternal metabolites (*N* = 1 with confirmed identity) and 11 cord blood metabolites (*N* = 5 with confirmed identity) were included in the LUCID analysis. 92 mother-newborn pairs with known parous history were assigned to two latent clusters by maternal parity and metabolomic signatures. As shown in Fig. 2 and Table S5, panel A, mother–newborn pairs of latent cluster 2 (*N* pairs = 32) had significantly higher birth weight Z-score (*p* < 0.001), and 31 (97%) in this cluster were multiparous. Maternal serum levels of sedoheptulose 7-phosphate and cord blood levels of glucose, fructose, progesterone, and 7alpha,25-dihydroxy-4-cholesten-3-one in the pathway of sugar metabolism were significantly lower in latent cluster 2 than in latent cluster 1, while maternal serum levels of glucose, L-4-hydroxyglutamate semialdehyde, succinate, Cys-Gly, ketoisovalerate, and 1-(1-alkenyl)-sn-glycero-3-phosphate in energy metabolism and amino acid metabolism pathways and cord blood levels of ketoleucine, L-serine, L-4-hydroxyglutamate semialdehyde, and lysophosphatidylcholine in amino acid metabolisms were significantly higher in latent cluster 2 (all *p*-values < 0.05).

Additionally, gestational weight gain was associated with 1 maternal metabolite and 2 cord blood metabolites with annotation (all *p*-values < 0.05) (Table S4). These three metabolomic signatures were then included in the LUCID analysis and the 96 mother-newborn pairs were assigned into two clusters (Fig. 2 and Table S5, panel B). Latent cluster 2, which clustered 31 mother-newborn pairs, had significant higher birth weight Z-scores and higher gestational weight gain (both *p*-values < 0.05). In the LUCID analysis model, maternal (S)-3-hydroxyisobutyrate and cord blood estradiol-17beta in pathways of energy metabolism and C21-steroid synthesis were significantly higher in latent cluster 2 than that in latent cluster1, while latent cluster 2 had significantly lower levels of cord blood N-acetyl-D-galactosamine in comparison with latent cluster 1 (all *p*-values < 0.05).

## Discussion

In the study, we applied high-resolution untargeted metabolomics to analyze maternal metabolomics at the 3rd trimester of pregnancy and newborn cord blood metabolomics. Dysregulation in the maternal pentose phosphate pathway, branched-chain amino acids degradation, and tryptophan, butanoate and glycerophospholipid metabolism, as well as dysregulation in newborn tryptophan metabolism, carnitine shuttle, and linoleate and branched-chain amino acid metabolism were associated with higher birth weight. Specifically, 22 maternal and 20 newborn metabolites were significantly associated with high birth weight. Higher maternal glucose and ketoisovalerate levels were associated with higher cord blood keto-acids involved in branched-chain amino acid metabolism. These metabolites also contributed to the association of maternal factors including parity and gestational weight gain, with higher birth weight and LGA risk (Fig. 2). In this study of 96 mother-newborn pairs, we found that maternal sugar and energy metabolism during pregnancy might affect newborn sugar metabolism, amino acid metabolism and C21-steroid hormone biosynthesis. These could further influence birth weight and play a role in the biological mechanisms linking maternal risk factors including parity and gestational weight gain on birth weight, especially LGA.

This study used xMWAS to reveal mother–newborn metabolomics network, and founded that higher maternal glucose and ketoisovalerate were associated with increased birth weight and newborn keto-acid (Table S1 and S3). Previous studies have similar findings that maternal glucose levels were positively associated with cord blood BCAAs and their metabolic by-products [32, 33]. Elevated BCAA levels, often observed in insulin resistance and diet-induced obesity, can lead to altered branched-chain keto-acid levels and LGA [34, 35]. An overload of BCAAs may induce insulin resistance, furthering metabolic disruption and morbidity risk [36, 37]. Ketoisovalerate has been shown to have inhibitory metabolic effects on gluconeogenesis and pyruvate dehydrogenase [38] and can also promote platelet aggregation and thrombosis [39]. Therefore, the findings of this study that fetal accumulation of branched-chain keto-acids in high birth weight newborns could signify impaired BCAA metabolism, insulin resistance, and amplified growth signaling, all of which predispose the newborn to further pathologies. Additionally, infant glucose, fructose, progesterone, and cortexolone were inversely associated with high birth weight and LGA risk. Downregulated progesterone levels in LGA have also been identified in other studies [9, 40]. These altered pathways could be a crucial mechanism linking a multiparous state to the risk of LGA in the newborn.

Gestational weight gain has long been considered to be a risk factor for LGA and macrosomia [41, 42]. Here, we applied LUCID model to investigate how maternal risk factors influence adverse birthweight outcomes through altering metabolomic signatures, and observed that altered maternal BCAA degradation and cord blood C21-steriod hormone biosynthesis and metabolism play a role in regulating the effects of gestational weight gain during pregnancy on infant birth weight (Fig. 2). Elevated maternal 3-hydroxyisobutryrate and infant estradiol-17beta were associated with high birth weight, which is consistent with previous studies [43–45]. A birth cohort study found that maternal urinary BCAAs, the BCAA degradation downstream byproduct 3-hydroxyisobutryrate, and steroid hormone by-products were associated with a significant fetal weight gain in the 3rd trimester [43].

A primary strength of this study is the prospective study design and comprehensive covariate data, as well as and the utilization of untargeted high-resolution metabolomics which enabled a thorough exploration of both maternal and neonatal metabolic pathways implicated in the connection between maternal factors and high birth weight. We identified the metabolomic profiles potentially associated with heightened vulnerability to the impacts of greater gestational weight gain and multiparity on high birth weight. We acknowledge that our study might have several limitations. Firstly, this research project leveraged an ongoing study with a relatively small sample size of maternal serum and cord blood pairs archived by mid-2019. Nevertheless, our study cohort, predominantly enrolled low-income Hispanic/Latina women, offers a unique opportunity to address the pathophysiological mechanisms of adverse birth outcomes in underrepresented populations. This study obtained both maternal and cord blood metabolomics, and the results from these different layers mutually reinforce each other. Secondly, untargeted metabolomics cannot provide absolute concentrations of metabolites and chemical annotations can pose challenges; however, we successfully annotated three maternal metabolites and eight cord blood metabolites with confirmed identity according to the rigorous criteria outlined by MSI at level 1. The remaining metabolomic features were validated by the advanced statistical methods of pathway analysis and network analysis through mapping metabolomic features to biological pathways and annotating them with confident prediction. Metabolomics studies can be used to identify etiology of high birth weight, and to develop future intervention strategy to reduce the risk of LGA as well as the risk of childhood obesity. Findings from our untargeted metabolomics analysis will guide the prioritization of metabolic pathways for further validation through targeted metabolomics approaches.

## Supplementary information

Supplementary material of Dysregulated Maternal and Newborn Fatty Acid, Sugar and Amino Acid Metabolism Associated with High Birth Weight

## Data Availability

The datasets generated during and/or analyzed during the current study are available from the corresponding author upon reasonable request.

## Code availability

The code for all statistical analyses is available from the corresponding author upon reasonable request.
