# Supplementary material for: Dysregulated maternal and newborn fatty acid, sugar and amino acid metabolism associated with high birth weight
Source: Int J Obes (Lond). 2025 Apr 17;49(7):1345–53. doi: 10.1038/s41366-025-01775-9 (PMC12283401; doi:10.1038/s41366-025-01775-9)
Supplement: Supplementary file 1 — Supplementary material of Dysregulated Maternal and Newborn Fatty Acid, Sugar and Amino Acid Metabolism Associated with High Birth Weight [file 41366_2025_1775_MOESM1_ESM.docx]

Supplementary material

**Method**

**Untargeted metabolomic profiles**

Before conducting high-resolution metabolomic analysis, cord blood plasma and maternal serum samples were taken out from storage at -80°C and thawed on ice. To eliminate any precipitate proteins, a sample of 50 μL was treated with 100 μL of ice-cold LC-MS grade acetonitrile, left to stabilize for 30 minutes on ice, and then spun in a centrifuge (16.1×*g* at 4 °C) for 10 minutes. Then, the sample was transferred to a 200 μL autosampler vial and kept at 4°C for analysis within 22 hours.

We used a dual pump configuration in the operating of chromatography system, allowing parallel separation of analytes and column flushing. For two high-resolution metabolomics methods – hydrophilic interaction liquid chromatography (HILIC) with positive electrospray ionization (ESI) and C18 hydrophobic reversed-phase chromatography with negative ESI – the mobile phase flow rate was initially set at 0.35 mL/min for the first 1.5 minutes, then increased to 0.5 mL/min and maintained for the final 4 minutes. The high-resolution mass spectrometer was operated at a resolution of 120,000 and within a mass-to-charge ratio (m/z) range of 85–1275. During untargeted data acquisition, no specific masses were selected for exclusion or inclusion, and data was only acquired in MS1 mode.

Before any statistical analysis, we conducted quality control, data normalization, and data transformation (as shown in **Figure S1)**. Firstly, metabolomic features with detection less than 50% were removed. After completeness check, our metabolomic data had 8639 features in the HILIC positive mode and 7268 features in the C18 negative mode from cord blood, as well as 8813 features in the HILIC positive mode and 5355 features in the C18 negative mode from 3^rd^ trimester maternal serum. Among the 96 cord blood plasma samples and 96 maternal serum samples, 191 samples had detected features > 60%, except one cord blood sample in HILIC positive mode with only 39.0% detected features (3,372 features). Missing metabolomic intensity data was imputed by half of the minimum intensities of each metabolomic feature. Quantile normalization and log_2_ transformation were further performed to normalize the data.^1^

The coefficients of variation (CVs) of the Qstd3 samples^2^ inserted in 5 analytical batches were calculated to control the data quality for untargeted metabolomic data. The CVs for cord blood were based on 16 Qstd3 samples, and the CVs for maternal serum were based on 14 Qstd3 samples. Before CV analyzing, all metabolomic features were log_2_ transformed to achieve similar scale with the metabolomic data of MADRES samples. The distribution of CVs for all features is presented in **Figure S2**. The medians (1^st^ quartile, 3^rd^ quartile) of CVs across 11770 detected HILIC positive features and 7268 detected C18 negative features were 0.04 (0.03, 0.06) and 0.03 (0.02, 0.05) for the 3 batches used to analyze cord blood samples. For maternal sample involved batches, the medians (1^st^ quartile, 3^rd^ quartile) of CVs across 11770 detected HILIC positive features and 7268 detected C18 negative features were 0.04 (0.02, 0.06) and 0.03 (0.02, 0.05). In order to control for potential batch effects, metabolomic features with CVs > 0.3 were excluded from all statistical analyses.

**Table S1.** Chemical annotations of maternal metabolomic features ^a^ that were associated with birth weight Z-score.

| **m/z** | **RT (sec)** | **Adduct** | **Chemical ID^b^** | **Metabolite** | **β** | **Method RT** | **Mummichog predicted pathways associated with birth weight Z-score** |
| --- | --- | --- | --- | --- | --- | --- | --- |
|  |  |  |  |  | **(p-value)** ^c^ |  |  |
| 219.027 | 77.1 | M+K[1+] | C00124 | Fructose | 0.77 (0.002) | HILIC+ | Tryptophan metabolism |
| 215.033 | 21 | M+Cl[-] | C00031 | Glucose | 0.78 (0.05) | C18- | Pentose phosphate pathway;Galactose metabolism;Glycosphingolipid biosynthesis - globoseries;Sialic acid metabolism;Fructose and mannose metabolism;N-Glycan Degradation; Glycerophospholipid metabolism |
| 199.038 | 21.9 | M+Cl[-] | C00586 | 2-Deoxyglucose | -0.41 (<0.001) | C18- | Fructose and mannose metabolism;N-Glycan Degradation |
| 1086.2555 | 69.2 | M+K[1+] | tmndnccoa | Timnodonyl coenzyme A | -1.56 (0.04) | HILIC+ | Fatty acid oxidation |
| 960.3081 | 69.4 | M-H2O+H[1+] | C02593 | Tetradecanoyl-CoA (n-C14:0CoA) | -1.53 (0.03) | HILIC+ | Fatty acid oxidation |
| 102.055 | 96.9 | M-HCOOH+H[1+] | C05938 | L-4-Hydroxyglutamate semialdehyde | 0.91 (0.01) | HILIC+ | Arachidonic acid metabolism;Tryptophan metabolism;Glutathione Metabolism |
|  |  | M-CO+H[1+] | C04282 | 1-Pyrroline-4-hydroxy-2-carboxylate |  |  |  |
| 321.2416 | 30.5 | M+H[1+] | CE5016 | 13,14-dihydroxy-retinol | 0.09 (0.04) | HILIC+ | Arachidonic acid metabolism;Vitamin A (retinol) metabolism |
|  |  | M-H2O+H[1+] | C14773 | 8,9-DHET |  |  |  |
| 104.0434 | 290 | M(C13)-H[-] | C06001 | (S)-3-Hydroxyisobutyrate | 1.06 (0.01) | C18- | Valine, leucine and isoleucine degradation;Butanoate metabolism |
| 184.0192 | 19.9 | M+Cl37[-] | C00025 | L-Glutamate | 0.35 (0.03) | C18- | Valine, leucine and isoleucine degradation;Butanoate metabolism |
| 161.0456 | 25.7 | M+HCOO[-] | C00141 | Ketoisovalerate | 0.77 (0.02) | C18- | Valine, leucine and isoleucine degradation;Butanoate metabolism |
|  |  | M+CH3COO[-] | C06002 | (S)-Methylmalonate semialdehyde |  |  |  |
| 176.0929 | 25.2 | M+CH3COO[-] | C00183 | L-Valine | 0.49 (0.02) | C18- | Valine, leucine and isoleucine degradation;Butanoate metabolism |
|  |  | M+HCOO[-] | C00407 | L-Isoleucine |  |  |  |
| 177.0405 | 283.4 | M+CH3COO[-] | C00042 | Succinate | 0.88 (0.02) | C18- | Valine, leucine and isoleucine degradation;Butanoate metabolism;Galactose metabolism;Sialic acid metabolism;Pentose phosphate pathway |
|  |  | M-H[-] | C00198 | D-Glucono-1,5-lactone |  |  |  |
|  |  | M-H2O-H[-] | C00257 | D-Gluconate |  |  |  |
| 161.0379 | 83.1 | M-H2O+H[1+] | C01419 | Cys-Gly | 0.69 (0.02) | HILIC+ | Glutathione Metabolism |
| 220.1008 | 95.6 | M(C13)+H[1+] | C03740 | 5-L-Glutamyl-L-alanine | 0.38 (0.03) | HILIC+ | Glutathione Metabolism |
| 115.039 | 285.2 | M-HCOOH+H[1+] | C00322 | 2-Oxoadipate | -0.22 (0.04) | HILIC+ | Tryptophan metabolism |
| 126.055 | 278.1 | M-CO+H[1+] | C00632 | 3-Hydroxyanthranilate | 0.19 (0.04) | HILIC+ | Tryptophan metabolism |
| 148.0393 | 281.5 | M-H2O+H[1+] | C05653 | N-Formylanthranilate | 0.29 (0.02) | HILIC+ | Tryptophan metabolism |
| 169.0761 | 24.1 | M+H[1+] | CE3087 | beta-carboline | 0.59 (0.03) | HILIC+ | Tryptophan metabolism |
| 180.0657 | 290.6 | M+H2O+H[1+] | C05637 | 4,8-Dihydroxyquinoline | 0.09 (0.05) | HILIC+ | Tryptophan metabolism |
| 182.0579 | 80.1 | M+Na[1+] | C00637 | Indole-3-acetaldehyde | -0.17 (0.01) | HILIC+ | Tryptophan metabolism |
| 179.0563 | 26.1 | M+HCOO[-] | C01801 | Deoxyribose | 0.92 (0.01) | C18- | Pentose phosphate pathway |
| 209.0667 | 25.8 | M+CH3COO[-] | C00508 | L-Ribulose | -0.43 (<0.001) | C18- | Pentose phosphate pathway |
|  |  | M-H[-] | C02076 | Sedoheptulose |  |  |  |
| 370.9554 | 30.4 | M+Br81[-] | C05382 | Sedoheptulose 7-phosphate | -0.49 (0.01) | C18- | Pentose phosphate pathway |
| 113.0244 | 25.3 | M-H2O-H[-] | C06010 | (S)-2-Acetolactate | 0.95 (0.01) | C18- | Butanoate metabolism |
| 161.0456 | 288.4 | M-H+O[-] | C06006 | (S)-2-Aceto-2-hydroxybutanoate | 0.91 (0.05) | C18- | Butanoate metabolism |
| 182.0752 | 291.6 | M(C13)-H[-] | C01697 | Galactitol | 0.70 (0.04) | C18- | Fructose and mannose metabolism;Galactose metabolism |
| 253.0929 | 25.7 | M-H[-] | C05401 | 3-beta-D-Galactosyl-sn-glycerol | -0.50 (0.001) | C18- | Galactose metabolism;Sialic acid metabolism;Glycerophospholipid metabolism |
| 358.2964 | 240.1 | M+CH3COO[-] | C00319 | Sphingosine | 0.08 (0.05) | C18- | Glycerophospholipid metabolism |
| 375.2299 | 244 | M-H2O-H[-] | C15646 | 1-(1-Alkenyl)-sn-glycero-3-phosphate | 0.19 (0.01) | C18- | Glycerophospholipid metabolism |
| 380.2554 | 274.5 | M-H[-] | C01120 | Sphinganine 1-phosphate | -0.66 (0.005) | C18- | Glycerophospholipid metabolism |
| 135.1168 | 25.8 | M+2H[2+] | CE5014 | Anhydroretinol | 0.48 (0.02) | HILIC+ | Vitamin A (retinol) metabolism |
| 387.1919 | 31.8 | M+HCOOK[1+] | C02075 | Retinyl ester | -0.65 (0.02) | HILIC+ | Vitamin A (retinol) metabolism |

^a^ Metabolomic features involved in statistically significant pathways identified by Mummichog pathway analysis to be associated with birth weight Z-score are presented in the table. The top three were features with confirmed chemical identities using MS2 spectra compared with authentic compounds according to the Metabolomics Standards Initiative (MSI) level 1 criteria.

^b^ Chemical IDs presented in the table are mostly KEGG ID, except tmndnccoa, CE5016, CE3087 and CE5014, which are BiGG IDs ( <http://bigg.ucsd.edu/>).

^c^ Linear regression was used to assess the associations between individual metabolomic feature and birth weight Z score adjusting for covariates including early vs late entry of women in the MADRES cohort, newborn’s Hispanic ethnicity, maternal age, marital status, maternal education status, household income, lifetime cigarette smoking, and mother’s recruitment site. Association estimates are interpreted as the increase or decrease in birth weight Z-score by one fold change in relative abundance of metabolomic feature among 96 mothers.

**Table S2.** Chemical annotations of cord blood metabolomic features ^a^ that were associated with birth weight Z-score.

| **m/z** | **RT (sec)** | **Adduct** | **Chemical ID^b^** | **Metabolite** | **β** | **Method RT** | **Mummichog predicted pathways associated with birth weight Z-score** |
| --- | --- | --- | --- | --- | --- | --- | --- |
|  |  |  |  |  | **(p-value)** ^c^ |  |  |
| 315.233 | 38.8 | M+H[1+] | C00410 | Progesterone/Tetrahydrocorticosterone/21-Hydroxypregnenolone | -0.39 (0.02) | HILIC+ | C21-steroid hormone biosynthesis and metabolism |
| 347.22 | 33.9 | M+H[1+] | C02140 | Cortexolone | -0.23 (0.02) | HILIC+ | C21-steroid hormone biosynthesis and metabolism |
| 311.296 | 290.9 | M-H[-] | C06425 | FA 20:0 (Arachidic acid) | -1.04 (0.03) | C18- | De novo fatty acid biosynthesis;Phytanic acid peroxisomal oxidation;Fatty acid activation |
| 115.04 | 25.2 | M-H[-] | C00141 | Oxovalerate/Ketoisovalerate | 0.63 (0.01) | C18- | Valine, leucine and isoleucine degradation |
| 129.056 | 25.4 | M-H[-] | C03465 | Ketoleucine/Ketoisoleucine | 0.74 (0.003) | C18- | Valine, leucine and isoleucine degradation |
| 219.027 | 77.1 | M+K[1+] | C00124 | Fructose | -0.44 (0.01) | HILIC+ | Tryptophan metabolism;Glycosphingolipid biosynthesis - globoseries;Glycosphingolipid biosynthesis - ganglioseries;Chondroitin sulfate degradation |
| 199.038 | 21.9 | M+Cl[-] | C00586 | 2-Deoxyglucose | **-0.60 (<0.001)** | C18- | N-Glycan Degradation |
| 215.033 | 21 | M+Cl[-] | C00031 | Glucose | -0.47 (0.05) | C18- | N-Glycan Degradation;Glycosphingolipid metabolism |
| 430.2961 | 50.8 | M-H2O-H[-] | C05462 | Chenodeoxyglycocholate | -0.10 (0.04) | C18- | Bile acid biosynthesis |
| 433.3327 | 293 | M-H[-] | C04554 | 3alpha,7alpha-Dihydroxy-5beta-cholestanate | -0.53 (0.02) | C18- | Bile acid biosynthesis |
|  |  | M-H2O-H[-] | CE4874 | 5beta-cholestane-3alpha,7alpha,12alpha,27,27-pentaol |  |  |  |
| 431.3171 | 276.8 | M-H[-] | CE2205 | 1alpha,24R,25-trihydroxyvitamin D3 | -0.54 (0.003) | C18- | Bile acid biosynthesis;Vitamin D3 (cholecalciferol) metabolism |
|  |  | M-H2O-H[-] | C04722 | 3alpha,7alpha,12alpha-Trihydroxy-5beta-cholestanoate |  |  |  |
| 475.3424 | 224.7 | M+CH3COO[-] | C17332 | 7alpha,25-Dihydroxy-4-cholesten-3-one | -0.4 (0.01) | C18- | Bile acid biosynthesis;Vitamin D3 (cholecalciferol) metabolism |
| 155.1067 | 287.8 | M+H[1+] | CE2577 | 4-oxo-2-nonenal | 0.29 (0.003) | HILIC+ | Linoleate metabolism |
|  |  | M-H2O+H[1+] | CE2576 | 4-hydroperoxy-2-nonenal |  |  |  |
| 158.0215 | 95.6 | M-H2O+H[1+] | C01041 | Monodehydroascorbate | 0.17 (0.01) | HILIC+ | Linoleate metabolism |
| 315.2527 | 30.9 | M+H2O+H[1+] | C14825 | 9(10)-EpOME | 0.15 (0.04) | HILIC+ | Linoleate metabolism |
| 331.2485 | 33.4 | M+H2O+H[1+] | C14827 | 9(S)-HPODE | 0.12 (0.03) | HILIC+ | Linoleate metabolism |
| 508.3762 | 49.3 | M+H[1+] | C04230 | Lysophosphatidylcholine | 0.64 (0.04) | HILIC+ | Linoleate metabolism |
| 201.1638 | 28.9 | M-C3H4O2+H[1+] | C00951 | Estradiol-17beta | 0.18 (0.01) | HILIC+ | C21-steroid hormone biosynthesis and metabolism |
| 230.1749 | 32.8 | M+H+Na[2+] | C05446 | 3alpha,7alpha,12alpha,26-Tetrahydroxy-5beta-cholestane | 0.14 (0.02) | HILIC+ | C21-steroid hormone biosynthesis and metabolism |
| 276.2045 | 213.3 | M(C13)-H[-] | C16300 | Stearidonic acid | -0.27 (0.04) | C18- | Omega-3 fatty acid metabolism |
| 313.1938 | 226 | M+Cl[-] | C06427 | (9Z,12Z,15Z)-Octadecatrienoic acid | -0.12 (0.03) | C18- | Omega-3 fatty acid metabolism;Fatty acid activation;De novo fatty acid biosynthesis |
| 1106.2743 | 83.9 | M+HCOONa[1+] | CE2439 | 3-oxo-6Z,9Z,12Z-octadecatrienoyl-CoA | 0.38 (0.02) | HILIC+ | Omega-6 fatty acid metabolism |
| 1030.2948 | 69.5 | M-H2O+H[1+] | tmndnccoa | Timnodonyl coenzyme A | 0.28 (0.03) | HILIC+ | Omega-6 fatty acid metabolism;Carnitine shuttle |
| 319.2225 | 254 | M+Cl37[-] | C00712 | (9Z)-Octadecenoic acid | -0.12 (0.01) | C18- | De novo fatty acid biosynthesis |
| 321.2383 | 275.4 | M+Cl37[-] | C01530 | Octadecanoate (n-C18:0) | -0.24 (0.02) | C18- | De novo fatty acid biosynthesis;Fatty acid activation |
| 201.1112 | 162.4 | M-NH3+H[1+] | pcrn | Propionyl-carnitine | 0.24 (0.03) | HILIC+ | Carnitine shuttle |
| 427.3608 | 32.9 | M(C13)+H[1+] | odecrn | Octadecenoyl carnitine | 0.53 (0.02) | HILIC+ | Carnitine shuttle |
| 428.373 | 32.8 | M+H[1+] | stcrn | Stearoylcarnitine | 0.54 (0.04) | HILIC+ | Carnitine shuttle |
| 494.324 | 49.6 | M+Na[1+] | c226crn | Cervonyl carnitine | 0.27 (0.05) | HILIC+ | Carnitine shuttle |
| 104.0434 | 290 | M(C13)-H[-] | C06001 | (S)-3-Hydroxyisobutyrate | -0.53 (0.02) | C18- | Valine, leucine and isoleucine degradation |
| 161.0092 | 18.5 | M-H+O[-] | C00026 | 2-Oxoglutarate | -0.24 (0.04) | C18- | Valine, leucine and isoleucine degradation;Phytanic acid peroxisomal oxidation |
| 136.0393 | 37.1 | M-C3H4O2+H[1+] | C01252 | 4-(2-Aminophenyl)-2,4-dioxobutanoate | -0.24 (0.02) | HILIC+ | Tryptophan metabolism |
| 154.0493 | 274.5 | M+H[1+] | C00632 | 3-Hydroxyanthranilate | 0.31 (0.05) | HILIC+ | Tryptophan metabolism |
| 169.0761 | 24.1 | M+H[1+] | CE3087 | beta-carboline | -0.38 (0.05) | HILIC+ | Tryptophan metabolism |
| 177.074 | 33.9 | M(C13)+H[1+] | C05634 | 5-Hydroxyindoleacetaldehyde | -0.06 (0.01) | HILIC+ | Tryptophan metabolism |
| 179.0899 | 33.3 | M(C13)+H[1+] | CE5629 | 1,2-dehydrosalsolinol | 0.14 (0.04) | HILIC+ | Tryptophan metabolism |
| 102.055 | 96.9 | M-HCOOH+H[1+] | C05938 | L-4-Hydroxyglutamate semialdehyde | 0.92 (0.003) | HILIC+ | Tryptophan metabolism;Arginine and Proline Metabolism; |
|  |  | M-CO+H[1+] | C04282 | 1-Pyrroline-4-hydroxy-2-carboxylate |  |  |  |
| 154.0479 | 94.4 | M+Na[1+] | C01165 | L-Glutamate 5-semialdehyde | 0.11 (0.01) | HILIC+ | Arginine and Proline Metabolism |
| 88.0631 | 157.1 | M+2H[2+] | C00062 | L-Arginine | -0.56 (0.01) | HILIC+ | Arginine and Proline Metabolism |
| 96.0443 | 278.7 | M-H2O+H[1+] | C03564 | 1-Pyrroline-2-carboxylate | -0.26 (0.03) | HILIC+ | Arginine and Proline Metabolism |
| 104.0353 | 21.6 | M-H[-] | C00065 | L-Serine | 0.79 (0.04) | C18- | Glycosphingolipid metabolism |
| 165.0558 | 128.1 | M-H2O-H[-] | C00588 | Choline phosphate | -0.21 (0.04) | C18- | Glycosphingolipid metabolism |
| 380.2554 | 274.5 | M-H[-] | C01120 | Sphinganine 1-phosphate | -0.71 (0.01) | C18- | Glycosphingolipid metabolism |
| 240.1074 | 28.2 | M+H2O+H[1+] | C01132 | N-Acetyl-D-galactosamine | -0.13 (0.03) | HILIC+ | Glycosphingolipid biosynthesis - globoseries;Glycosphingolipid biosynthesis - ganglioseries;Chondroitin sulfate degradation |

^a^ Metabolomic features involved in statistically significant pathways identified by Mummichog pathway analysis to be associated with birth weight Z-score are presented in the table. The top eight were features with confirmed chemical identities using MS2 spectra compared with authentic compounds according to the MSI level 1 criteria.

^b^ Chemical IDs presented in the table are mostly KEGG ID, except CE4874, CE2205, CE2577, CE2576, CE2439, tmndnccoa, pcrn, odecrn, stcrn, c226crn, CE3087 and CE5629, which are BiGG IDs ( <http://bigg.ucsd.edu/>).

^c^ Linear regression was used to assess the associations between individual metabolomic feature and birth weight Z score adjusting for covariates including early vs late entry of women in the MADRES cohort, newborn’s Hispanic ethnicity, maternal age, marital status, maternal education status, household income, lifetime cigarette smoking, mother’s recruitment site, and sample processing time of cord blood (≤3hrs vs >3hrs). Association estimates are interpreted as the increase or decrease in birth weight Z-score by one fold change in relative abundance of metabolomic feature among 96 newborns. Significant association with FDR value<0.2 was bolded.

**Table S3.** Degree weight centrality measure^a^ that indicated the importance of each maternal and newborn metabolomic signature in the entire network.

| **Cluster** | **Sample Source** | **Name** | **Centrality** |
| --- | --- | --- | --- |
| 1 | Third trimester maternal serum | (S)-2-Aceto-2-hydroxybutanoate | **0.73** |
|  |  | 3-Hydroxyanthranilate | 0.00 |
|  | Cord blood | beta-carboline | 0.16 |
|  |  | FA 20:0 (Arachidic acid) | 0.00 |
|  |  | Octadecanoate (n-C18:0) | 0.00 |
|  |  | Sphinganine 1-phosphate | 0.00 |
| 2 | Third trimester maternal serum | Sphinganine 1-phosphate | **1.00** |
|  |  | Fructose | **0.73** |
|  |  | Glucose | **0.43** |
|  |  | Ketoisovalerate/(S)-Methylmalonate semialdehyde | **0.22** |
|  |  | Deoxyribose | 0.16 |
|  |  | (S)-2-Acetolactate | 0.00 |
|  | Cord blood | Oxovalerate/Ketoisovalerate | **0.95** |
|  |  | Fructose | 0.08 |
|  |  | 5-Hydroxyindoleacetaldehyde | 0.00 |
|  |  | Glucose | 0.00 |
|  |  | (9Z,12Z,15Z)-Octadecatrienoic acid | 0.00 |
|  |  | L-Arginine | 0.00 |
|  |  | Ketoleucine/Ketoisoleucine | 0.00 |
|  |  | 4-(2-Aminophenyl)-2,4-dioxobutanoate | 0.00 |
| 3 | Third trimester maternal serum | Sedoheptulose 7-phosphate | **0.42** |
|  |  | Tetradecanoyl-CoA (n-C14:0CoA) | **0.29** |
|  |  | 4,8-Dihydroxyquinoline | 0.00 |
|  | Cord blood | 1alpha,24R,25-trihydroxyvitamin D3/3alpha,7alpha,12alpha-Trihydroxy-5beta-cholestanoate | **0.90** |
|  |  | Progesterone/Tetrahydrocorticosterone/21-Hydroxypregnenolone | 0.16 |
|  |  | 3alpha,7alpha-Dihydroxy-5beta-cholestanate/5beta-cholestane-3alpha,7alpha,12alpha,27,27-pentaol | 0.01 |
|  |  | N-Acetyl-D-galactosamine | 0.00 |
|  |  | 9(10)-EpOME | 0.00 |
| 4 | Third trimester maternal serum | 2-Deoxyglucose | **0.37** |
|  |  | L-Ribulose/Sedoheptulose | **0.36** |
|  |  | 3-beta-D-Galactosyl-sn-glycerol | 0.00 |
|  | Cord blood | Stearoylcarnitine | **0.83** |
|  |  | Octadecenoyl carnitine | **0.83** |
|  |  | Cortexolone | 0.16 |
|  |  | 2-Deoxyglucose | 0.00 |
|  |  | 1-Pyrroline-2-carboxylate | 0.00 |
| 5 | Third trimester maternal serum | 2-Oxoadipate | 0.00 |
|  | Cord blood | L-4-Hydroxyglutamate semialdehyde/1-Pyrroline-4-hydroxy-2-carboxylate | 0.00 |

^a^ Degree weight centrality measure (DWCM), which ranged from 0 to 1, was calculated based on the sum of a node’s absolute weights of connections. Higher DWCM indicates stronger connections in the network. Features with DWCM>0.2 were in bold font.

**Table S4.** Associations ^a^ of parity categories and gestational weight gain with 76 targeted metabolites.

| **Metabolomic Signatures** | **Parity** |  | **Gestational weight gain** |
| --- | --- | --- | --- |
|  | **β (p-value)** |  | **β (p-value)** |
| **Third trimester maternal metabolites** |  |  |  |
| L-4-Hydroxyglutamate semialdehyde/1-Pyrroline-4-hydroxy-2-carboxylate | 0.23 (**0.01**) |  | 4.43E-03 (0.43) |
| (S)-3-Hydroxyisobutyrate | 0.11 (0.19) |  | 1.22E-02 (**0.02**) |
| Timnodonyl coenzyme A | -0.04 (0.36) |  | -1.10E-03 (0.69) |
| (S)-2-Acetolactate | 0.14 (0.13) |  | 7.32E-03 (0.18) |
| 2-Oxoadipate | -0.02 (0.96) |  | -2.14E-02 (0.27) |
| 3-Hydroxyanthranilate | 0.87 (**0.02**) |  | 2.59E-02 (0.24) |
| Anhydroretinol | 0.17 (0.32) |  | 1.23E-02 (0.21) |
| N-Formylanthranilate | 0.38 (0.16) |  | 1.44E-02 (0.37) |
| Cys-Gly | 0.30 (**0.01**) |  | 5.73E-03 (0.42) |
| Ketoisovalerate/(S)-Methylmalonate semialdehyde | 0.26 (**0.01**) |  | 9.19E-03 (0.14) |
| (S)-2-Aceto-2-hydroxybutanoate | 0.19 (**0.01**) |  | 3.04E-03 (0.50) |
| beta-carboline | 0.12 (0.34) |  | 4.84E-03 (0.52) |
| L-Valine/L-Isoleucine | 0.14 (0.41) |  | 1.97E-02 (0.05) |
| Succinate/D-Glucono-1,5-lactone/D-Gluconate | 0.20 (**0.03**) |  | 9.64E-03 (0.08) |
| Deoxyribose | 0.16 (0.11) |  | 6.96E-03 (0.25) |
| 4,8-Dihydroxyquinoline | 0.78 (0.29) |  | 2.73E-02 (0.55) |
| Indole-3-acetaldehyde | -1.01 (0.07) |  | -6.43E-02 (0.05) |
| Galactitol | 0.04 (0.69) |  | 4.07E-03 (0.47) |
| L-Glutamate | 0.05 (0.82) |  | 1.00E-02 (0.44) |
| 2-Deoxyglucose | -0.35 (0.24) |  | -1.77E-02 (0.32) |
| L-Ribulose/Sedoheptulose | -0.42 (0.19) |  | -1.87E-02 (0.31) |
| Glucose | 0.20 (**0.03**) |  | 1.01E-02 (0.06) |
| Fructose | 0.24 (0.07) |  | -3.89E-03 (0.63) |
| 5-L-Glutamyl-L-alanine | 0.04 (0.82) |  | 3.62E-03 (0.76) |
| 3-beta-D-Galactosyl-sn-glycerol | -0.36 (0.11) |  | -1.36E-02 (0.31) |
| 13,14-dihydroxy-retinol/8,9-DHET | -0.54 (0.49) |  | 2.52E-02 (0.59) |
| Sphingosine | -0.28 (0.76) |  | 3.44E-02 (0.53) |
| Sedoheptulose 7-phosphate | -0.39 (**0.04**) |  | -8.01E-03 (0.47) |
| 1-(1-Alkenyl)-sn-glycero-3-phosphate | 1.63 (**<0.001**) |  | 6.17E-03 (0.83) |
| Sphinganine 1-phosphate | -0.15 (0.31) |  | -3.27E-04 (0.97) |
| Retinyl ester | -0.11 (0.37) |  | -9.51E-03 (0.19) |
| Tetradecanoyl-CoA (n-C14:0CoA) | -0.05 (0.28) |  | -2.11E-03 (0.47) |
| **Cord blood metabolites** |  |  |  |
| L-4-Hydroxyglutamate semialdehyde/1-Pyrroline-4-hydroxy-2-carboxylate | 0.32 (**0.01**) |  | 1.10E-02 (0.11) |
| Timnodonyl coenzyme A | 0.26 (0.36) |  | -3.95E-03 (0.81) |
| L-Serine | 0.25 (**0.01**) |  | 2.91E-03 (0.60) |
| (S)-3-Hydroxyisobutyrate | -0.16 (0.34) |  | 3.90E-03 (0.68) |
| 3-oxo-6Z,9Z,12Z-octadecatrienoyl-CoA | -0.06 (0.79) |  | 1.38E-02 (0.31) |
| Oxovalerate/Ketoisovalerate | 0.25 (0.12) |  | -6.43E-03 (0.49) |
| Ketoleucine/Ketoisoleucine | 0.30 (**0.03**) |  | -5.35E-03 (0.53) |
| 4-(2-Aminophenyl)-2,4-dioxobutanoate | 0.21 (0.58) |  | -2.62E-02 (0.22) |
| L-Glutamate 5-semialdehyde | 1.72 (0.06) |  | 2.28E-02 (0.67) |
| 3-Hydroxyanthranilate | -0.15 (0.54) |  | 9.29E-04 (0.95) |
| 4-oxo-2-nonenal/4-hydroperoxy-2-nonenal | 0.70 (0.07) |  | 1.38E-03 (0.95) |
| Monodehydroascorbate | 0.61 (0.30) |  | 2.78E-02 (0.40) |
| 2-Oxoglutarate | -0.49 (0.12) |  | 8.16E-03 (0.65) |
| Choline phosphate | -0.62 (0.09) |  | -8.81E-03 (0.67) |
| beta-carboline | -0.05 (0.79) |  | -5.01E-03 (0.65) |
| 5-Hydroxyindoleacetaldehyde | -0.99 (0.51) |  | -8.77E-02 (0.31) |
| 1,2-dehydrosalsolinol | 1.15 (**0.02**) |  | 1.46E-02 (0.61) |
| 2-Deoxyglucose | -0.19 (0.39) |  | -2.14E-02 (0.08) |
| Propionyl-carnitine | 0.79 (**0.02**) |  | -4.17E-05 (1.00) |
| Estradiol-17beta | 0.72 (0.17) |  | 7.54E-02 (**0.01**) |
| Glucose | -0.44 (**0.004**) |  | 6.95E-03 (0.44) |
| Fructose | -0.76 **(<0.001**) |  | 2.98E-03 (0.81) |
| 3alpha,7alpha,12alpha,26-Tetrahydroxy-5beta-cholestane | 0.42 (0.5) |  | 1.29E-02 (0.71) |
| N-Acetyl-D-galactosamine | -0.64 (0.31) |  | -9.88E-02 (**0.004**) |
| Stearidonic acid | 0.03 (0.92) |  | -1.45E-02 (0.38) |
| FA 20:0 (Arachidic acid) | 0.003 (0.96) |  | 5.05E-03 (0.24) |
| (9Z,12Z,15Z)-Octadecatrienoic acid | -0.33 (0.61) |  | 5.90E-03 (0.88) |
| Progesterone/Tetrahydrocorticosterone/21-Hydroxypregnenolone | -0.53 (**0.01**) |  | 2.65E-03 (0.83) |
| 9(10)-EpOME | 0.67 (0.16) |  | 2.95E-02 (0.29) |
| (9Z)-Octadecenoic acid | -0.43 (0.55) |  | -4.41E-02 (0.30) |
| Octadecanoate (n-C18:0) | -0.10 (0.78) |  | -1.42E-02 (0.49) |
| 9(S)-HPODE | 0.12 (0.87) |  | -2.85E-02 (0.48) |
| Cortexolone | -0.95 (**0.01**) |  | 5.25E-03 (0.81) |
| Sphinganine 1-phosphate | -0.18 (0.20) |  | -5.19E-03 (0.51) |
| Octadecenoyl carnitine | 0.04 (0.82) |  | -4.45E-03 (0.65) |
| Stearoylcarnitine | -0.03 (0.83) |  | -2.14E-03 (0.81) |
| Chenodeoxyglycocholate | 1.01 (0.18) |  | -1.68E-02 (0.70) |
| 1alpha,24R,25-trihydroxyvitamin D3/3alpha,7alpha,12alpha-Trihydroxy-5beta-cholestanoate | -0.14 (0.50) |  | -2.21E-02 (0.06) |
| 3alpha,7alpha-Dihydroxy-5beta-cholestanate/5beta-cholestane-3alpha,7alpha,12alpha,27,27-pentaol | 0.05 (0.78) |  | -1.78E-02 (0.06) |
| 7alpha,25-Dihydroxy-4-cholesten-3-one | -0.71 (**0.003**) |  | -4.58E-03 (0.75) |
| Cervonyl carnitine | -0.08 (0.76) |  | -1.21E-02 (0.43) |
| Lysophosphatidylcholine | 0.35 (**0.003**) |  | -1.25E-03 (0.86) |
| L-Arginine | 0.14 (0.41) |  | 1.33E-02 (0.17) |
| 1-Pyrroline-2-carboxylate | 0.16 (0.63) |  | 1.27E-03 (0.95) |

^a^ Linear regression was used to evaluate the association of parity categories with 76 targeted metabolites, after adjusting for covariates. Models analyzing maternal metabolites as the outcomes were adjusted for early vs late entry group of women enrolled in the MADRES cohort, maternal Hispanic ethnicity, maternal age, maternal education status, marital status, household income, lifetime cigarette smoking, gestational age at sample collection and mother’s recruitment site. Models analyzing cord blood metabolites as the outcomes were adjusted for early vs late entry group of women enrolled in the MADRES cohort, neonatal Hispanic ethnicity, newborn sex, maternal age, maternal education status, marital status, household income, lifetime cigarette smoking, gestational age at delivery, mother’s recruitment site, and sample processing time of cord blood (≤3hrs vs >3hrs). Linear association estimate β and corresponding p-values are presented. Significant associations were in bold font (p<0.05). The association estimates for parity are interpreted as the increase or decrease in metabolomic feature for 2^nd^ or later born comparing to first born. The association estimates for gestational weight gain are interpreted as the increase or decrease in metabolomic feature by per 1kg increase in gestational weight gain.

**Table S5.** Characteristics of 96 MADRES mother-newborn pairs across two latent clusters assigned by latent unknown clustering with integrated data (LUCID). Panel a presents the distribution of parity categories and birth weight categories by two latent clusters from LUCID. Panel b presents the distribution of maternal weight gain and birth weight categories by two latent clusters assigned by LUCID.

(A)

|  | Latent Cluster-1  N (%) | Latent Cluster-2  N (%) | p-value |
| --- | --- | --- | --- |
| **Parity**^a^ |  |  | **<0.001**^b^ |
| First-born | 30 (97) | 1 (3) |  |
| Second-born or later | 30 (49) | 31 (51) |  |
| **Birth weight category** |  |  | **<0.001**^b^ |
| AGA (Appropriate for Gestational Age) | 50 (70) | 21 (30) |  |
| SGA (Small for Gestational Age) | 8 (100) | 0 (0) |  |
| LGA (Large for Gestational Age) | 2 (15) | 11(85) |  |
| **Birth weight Z-score**^c^ | -0.34 (0.91) | 0.95 (1.05) | **<0.001**^d^ |

(B)

|  | Latent Cluster-1  N (%) | Latent Cluster-2  N (%) | p-value |
| --- | --- | --- | --- |
| **Birth weight category** |  |  | **<0.001**^b^ |
| AGA (Appropriate for Gestational Age) | 57 (76) | 18 (24) |  |
| SGA (Small for Gestational Age) | 7 (88) | 1 (13) |  |
| LGA (Large for Gestational Age) | 1 (8) | 12 (92) |  |
| **Gestational weight gain**^c^ | 9.41 (6.52) | 17.13 (5.67) | **<0.001**^d^ |
| **Birth weight Z-score**^c^ | -0.29 (0.91) | 0.88 (1.12) | **<0.001**^d^ |

^a^ Four mother-newborn pairs were excluded due to parity unknown.

^b^ Fisher’s exact test was used to evaluate the association. Supervised clustering approach was used to assign the two latent clusters.

^c^ This variable is presented as mean (standard deviation) rather than N (%).

^d^ Two-Sample T-test was used to evaluate the difference in mean birth weight Z-score between two clusters. The two clusters were assigned by supervised clustering approach.

**Figure S1.** The flow chart of quality control, data normalization and data transformation for metabolomic features.

**
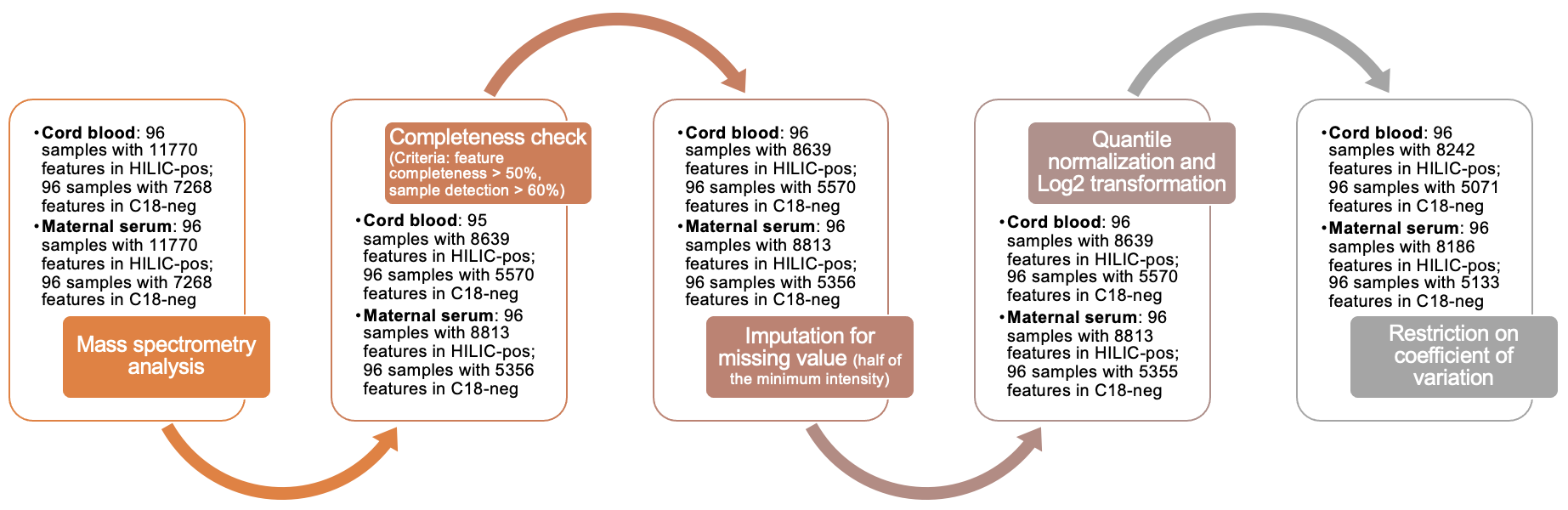
**

**Figure S2.** Distributions of coefficients of variation (CVs) among Qstd3 samples for untargeted metabolomics data.


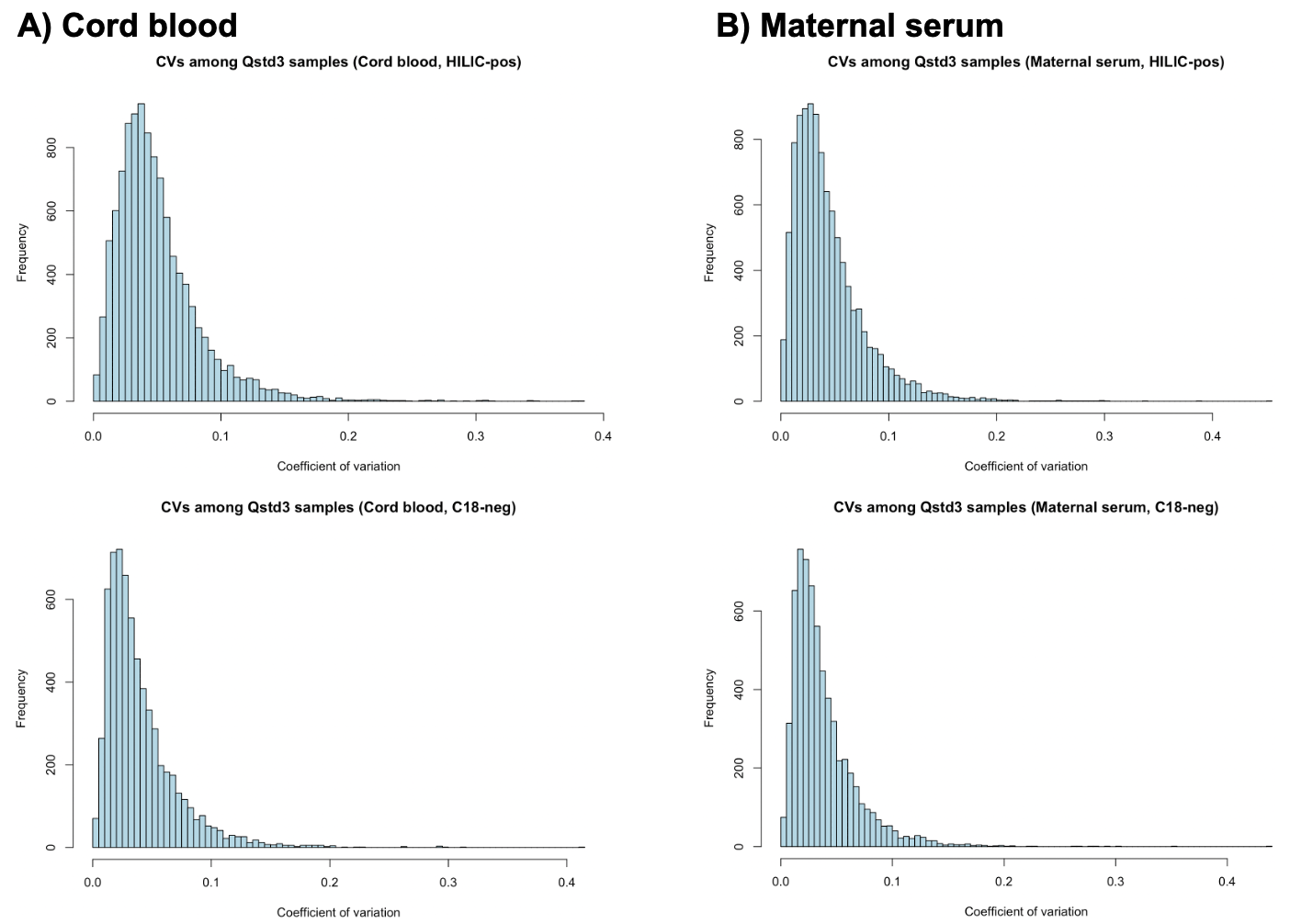


**Figure S3.** Metabolome-wide associations between metabolomic features with CVs among Qstd3 samples < 0.3 and birth weight Z-score in 96 newborns.


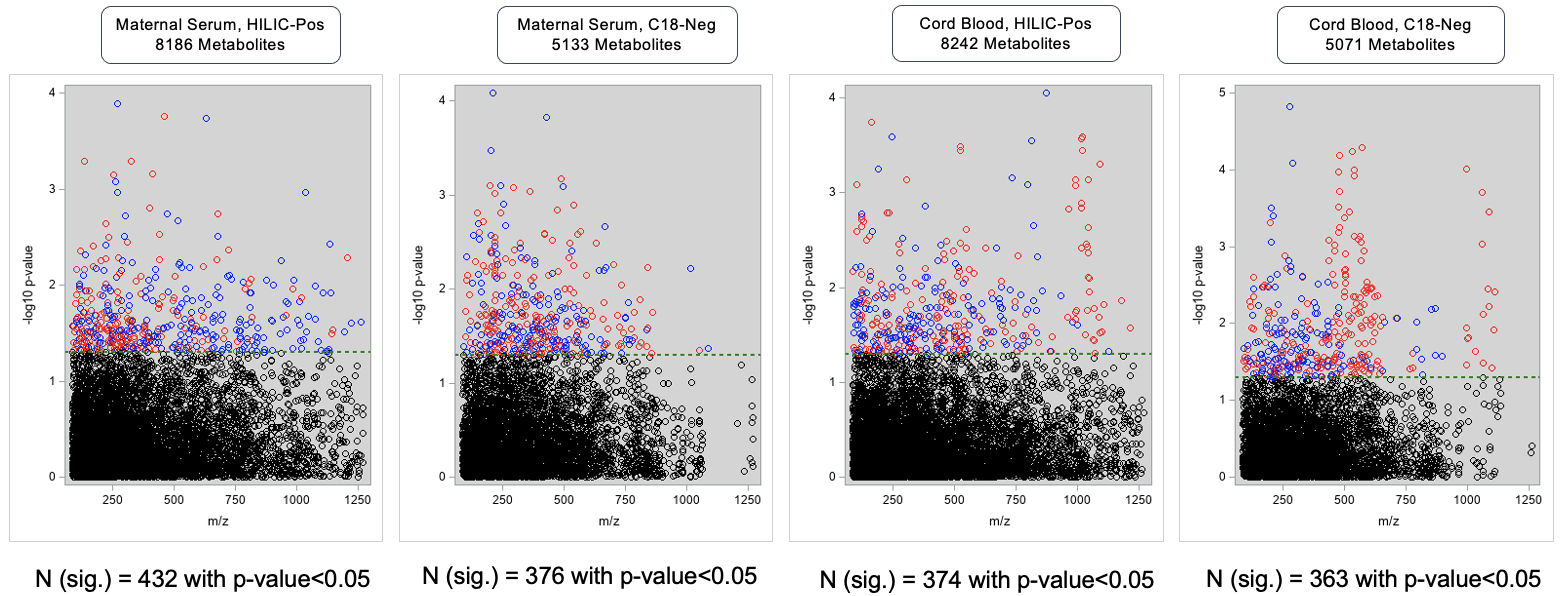


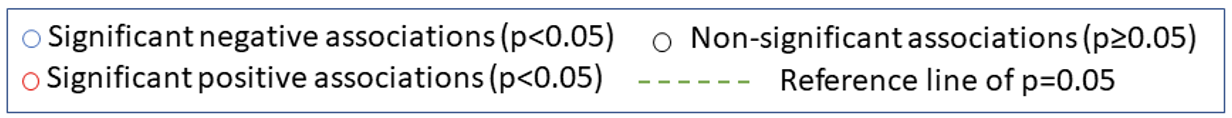


**Figure S4**. Integrated network analysis using annotated metabolomic features from 96 cord blood plasma samples and 96 3rd trimester maternal serum samples.


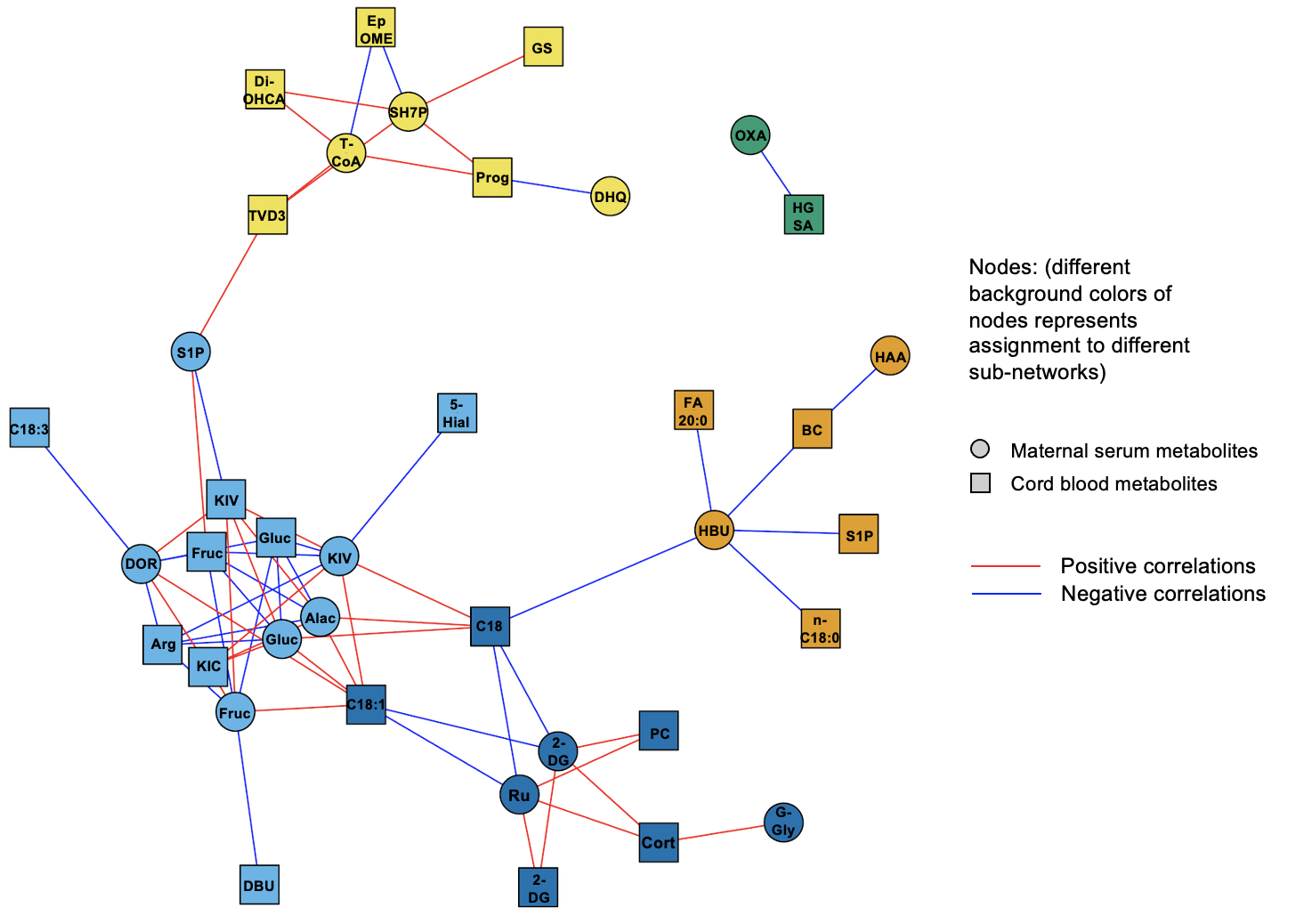


The xMWAS integrated network analysis was performed after controlling for covariates including early vs late entry groups of women enrolled in the MADRES cohort, recruitment site, maternal age at delivery, Hispanic ethnicity, gestational age at time of sample collection, household income, maternal marital status, maternal education, maternal smoking history, and sample processing time (within 3 hours or longer than 3 hours). Each node represents a metabolite. Nodes in circle shapes represent maternal metabolites and nodes in square shapes represent newborn metabolites. Metabolic communities are clustered into sub-networks and are classified by different colors. Maternal serum metabolomic features: (S)-2-Aceto-2-hydroxybutanoate=HBU, 3-Hydroxyanthranilate=HAA, Sphinganine 1-phosphate=S1P, Fructose=Fruc, Glucose=Gluc, Ketoisovalerate/(S)-Methylmalonate semialdehyde=KIV, Deoxyribose=DOR, (S)-2-Acetolactate=Alac, 2-Oxoadipate=OXA, Sedoheptulose 7-phosphate=SH7P, Tetradecanoyl-CoA (n-C14:0CoA)=T-CoA, 4,8-Dihydroxyquinoline=DHQ, 2-Deoxyglucose=2-DG, L-Ribulose/Sedoheptulose=Ru, 3-beta-D-Galactosyl-sn-glycerol=G-Gly; Cord blood metabolomic features: beta-carboline=BC, FA 20:0 (Arachidic acid)=FA 20:0, Octadecanoate (n-C18:0)=n-C18:0, Sphinganine 1-phosphate=S1P, Oxovalerate/Ketoisovalerate=KIV, Fructose=Fruc, 5-Hydroxyindoleacetaldehyde=5-Hial, Glucose=Gluc, (9Z,12Z,15Z)-Octadecatrienoic acid=C18:3, L-Arginine=Arg, Ketoleucine/Ketoisoleucine=KIC, 4-(2-Aminophenyl)-2,4-dioxobutanoate=DBU, L-4-Hydroxyglutamate semialdehyde/1-Pyrroline-4-hydroxy-2-carboxylate=HGSA, 1alpha,24R,25-trihydroxyvitamin D3/3alpha,7alpha,12alpha-Trihydroxy-5beta-cholestanoate=TVD3, Progesterone/Tetrahydrocorticosterone/21-Hydroxypregnenolone=Prog, 3alpha,7alpha-Dihydroxy-5beta-cholestanate/5beta-cholestane-3alpha,7alpha,12alpha,27,27-pentaol=Di-OHCA, N-Acetyl-D-galactosamine=GS, 9(10)-EpOME=EpOME, Stearoylcarnitine=C18, Octadecenoyl carnitine=C18:1, Cortexolone=Cort, 2-Deoxyglucose=2-DG, 1-Pyrroline-2-carboxylate=PC.

**References**

1. Lee J, Park J, Lim MS, Seong SJ, Seo JJ, Park SM, et al. Quantile normalization approach for liquid chromatography-mass spectrometry-based metabolomic data from healthy human volunteers. *Anal Sci*. 2012;28(8):801-805.

2. Liu KH, Nellis M, Uppal K, Ma C, Tran V, Liang Y, et al. Reference Standardization for Quantification and Harmonization of Large-Scale Metabolomics. *Anal Chem*. 2020;92(13):8836-8844.
